# Estimating cognitive workload in robot assisted surgery using time and frequency features from EEG epochs with random forest regression

**DOI:** 10.1038/s41598-026-35986-5

**Published:** 2026-02-06

**Authors:** Mohammed Atheef G A, Omkar S Powar

**Affiliations:** Manipal Institute of Technology, Manipal Academy of Higher Education, Manipal, 576104 Karnataka India

**Keywords:** Cognitive workload (CW), Electroencephalography (EEG), Robot-assisted surgery (RAS), Feature extraction (FE), Power spectral density (PSD), Machine learning (ML), Random forest regressor (RFR), Biomedical engineering, Translational research

## Abstract

Cognitive workload (CW) refers to the mental effort required to perform a task and is critical to monitor in high-stakes environments such as robot-assisted surgery (RAS), where excessive demand can impair decision-making and performance. This study proposes a machine learning framework to estimate CW using electroencephalography (EEG) signals, focusing on four cortical regions: frontal, temporal, parietal, and occipital. EEG epochs were processed to extract both time-domain features (mean, variance, skewness, kurtosis, RMS, zero-crossings) and frequency-domain features (power spectral density across delta, theta, alpha, beta, and gamma bands). To enhance computational efficiency, data were downsampled from 500 to 128 Hz, with minimal signal degradation confirmed via topographic and spectrogram-based comparisons. Random Forest Regressor (RFR) was trained to predict region-specific EEG-derived CW scores, achieving high accuracy with R^2^ (coefficient of determination) values of 0.9947 (temporal), 0.9692 (parietal), 0.9635 (occipital), and 0.9329 (frontal), alongside low RMSE scores. Feature importance analysis identified kurtosis, RMS, and select power bands as key predictors. Model robustness was validated using tenfold cross-validation and statistical significance testing (p < 0.0001). Comparative evaluation with SVR, Linear Regression, and XGBoost confirmed the superior generalizability of the RFR model. Topographic EEG maps and time–frequency spectrograms visually supported region-specific activation patterns, reinforcing the effectiveness of spatially localized workload modeling. These findings demonstrate a promising, interpretable, and high-performing pipeline for EEG-based cognitive workload estimation, with broad implications for adaptive neuroergonomic systems in surgical and clinical settings.

## Introduction

The integration of Artificial Intelligence (AI) and Machine Learning (ML) into Robot-Assisted Surgery (RAS) has transformed modern surgical practices by enhancing precision, reducing invasiveness, and accelerating postoperative recovery. Despite these advancements, RAS presents unique cognitive challenges for surgeons, particularly in terms of CW a multifaceted construct that reflects the mental effort required to perform a task. CW plays a pivotal role in determining surgical performance, error likelihood, and overall patient outcomes, thereby underscoring its importance as a research focus^1,2^. Unlike traditional procedures, RAS requires surgeons to continuously interpret complex visual and haptic feedback and make high-stakes decisions in real-time, often through indirect robotic control interfaces. This intensifies cognitive demands and can contribute to cognitive fatigue, which may compromise task performance^3^. As a result, there is increasing interest in developing objective, continuous, and physiologically grounded methods for CW assessment. Among these, EEG has emerged as a promising tool, offering high temporal resolution and the ability to monitor neural activity in real-time, thereby enabling more precise and dynamic measurement of cognitive effort^4,5^.

EEG provides high temporal resolution for capturing cerebral activity and enables detailed investigation of neural oscillations across canonical frequency bands Delta (0.5–4 Hz), Theta (4–8 Hz), Alpha (8–13 Hz), Beta (13–30 Hz), and Gamma (30–50 Hz)^6^. These frequency-specific dynamics are widely recognized as biomarkers for distinct cognitive processes, including attention, memory load, and decision-making. EEG-derived features such as PSD, approximate entropy, and Event-Related Potentials (ERPs) offer valuable insights into cognitive workload states^7,8^. PSD serves as a core frequency-domain feature, reflecting the distribution of signal power across different frequency bands and its modulation in response to mental effort^9,10^. Historically, statistical and machine learning models such as LASSO regression and Random Forest have been employed to analyze EEG features. While useful, traditional models often assume linear relationships and may fail to capture the nonlinear and dynamic characteristics of neurophysiological signals. To address these limitations, signal processing techniques such as Welch’s method have been widely adopted to estimate PSD with improved robustness. By averaging overlapping segments of the EEG signal, Welch’s method reduces spectral variance and enhances feature reliability^11–16^. Accurate modeling of cognitive workload thus necessitates robust feature engineering that combines precise signal processing techniques with informative neurophysiological descriptors, ultimately improving the interpretability and performance of machine learning models.

Time-domain features such as mean, variance, root mean square (RMS), and skewness provide a compact yet informative summary of EEG amplitude fluctuations and waveform morphology, enabling the detection of underlying neural states. When combined with frequency-domain features such as power spectral densities, these descriptors create a multimodal feature space ideal for machine learning applications^17,18^. RFR, an ensemble-based learning algorithm, is particularly well-suited for modeling such high-dimensional physiological datasets. RFR is robust to multicollinearity, captures nonlinear dependencies, and is inherently resistant to overfitting an essential property when working with variable and noisy EEG signals^11,19–24^. Moreover, the model’s built-in feature importance analysis facilitates interpretability, which is critical in neurocognitive research. In this study, advanced feature selection techniques, including mutual information and recursive feature elimination (RFE), were employed to retain only the most discriminative EEG features. This process not only reduced computational overhead but also enhanced model generalizability and interpretability, making it feasible for real-time applications.

**Fig. 1:**
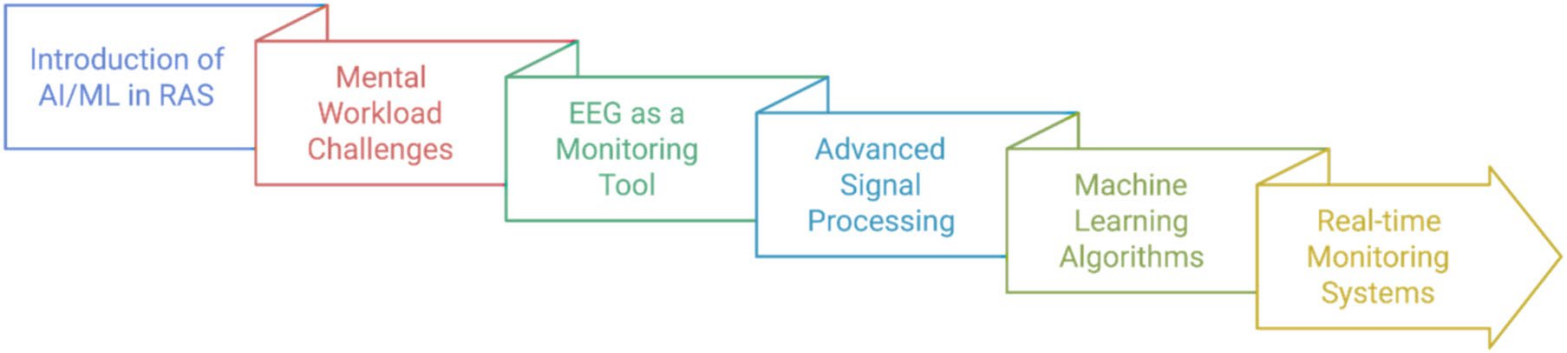
Integration of machine learning in robot assisted surgery.

The role of artificial intelligence (AI) and machine learning (ML) in robot-assisted surgery (RAS) extends beyond cognitive state estimation. As illustrated in Fig. 1, the integration of ML frameworks into RAS workflows holds transformative potential. Real-time cognitive workload monitoring systems can enable adaptive robotic interfaces that dynamically respond to the surgeon’s mental state modifying interface complexity, sensory feedback, or procedural pacing to optimize human–robot interaction. Such closed-loop systems are crucial for minimizing cognitive overload, reducing fatigue, and maintaining peak surgical performance. Additionally, ML-driven EEG analytics can revolutionize surgical education by providing objective cognitive metrics for skill assessment, performance benchmarking, and adaptive feedback delivery^25–30^. The present work builds upon the foundational efforts employed functional brain network metrics for cognitive workload estimation^2,31,32^. In contrast, our approach focuses on robust signal processing and interpretable machine learning using PSD and statistical features, offering computational advantages and feasibility. This fusion of EEG neuroscience, machine learning, and robotic technology propels the development of intuitive, adaptive surgical systems designed to enhance surgical safety, efficacy, and personalized training^33^.

## Literature review

Cognitive workload refers to the mental effort required to perform a task, often quantified using behavioral, physiological, or neurological indicators. Among these, EEG has gained significant attention for CW assessment due to its non-invasive, real-time capability and sensitivity to changes in cortical activation. Several studies have leveraged EEG signals to classify CW. Aricò et al. developed a passive brain-computer interface (BCI) to classify mental workload using EEG-derived features, achieving ~ 80% accuracy^34–41^. However, the model was task-specific and lacked generalizability. Borghini et al. employed SVM and K-Nearest Neighbour (KNN) classifiers on EEG features to achieve ~ 85% classification accuracy, though limited dataset diversity affected generalizability^40^. Applied machine learning algorithms such as SVM and Random Forest for CW classification, achieving accuracies of 88% and 92%, respectively^7,15,19,42–44^. These studies highlight the utility of EEG power spectral features (e.g., delta to gamma bands) for workload estimation.

In surgical contexts, Hashimoto et al. applied ML techniques to assess surgical performance, reporting an R^2^ of 0.89, though their work did not directly target CW^45^. Shadpour et al. modeled EEG-based surgical performance using Generalized Linear Mixed Models (GLMM) and LASSO regression, reaching R^2^ = 0.97^2^. However, their reliance on high-density EEG and extensive preprocessing limited real-time application. Lin et al. used LSTM networks to predict workload from EEG time series (~ 88% accuracy)^46^, but deep learning’s demand for large datasets and computational power makes it less suitable for real-time deployment. While past approaches have shown promising results, they often depend on high-dimensional feature sets, complex neural architectures, or high-density EEG systems^47,48^. These factors introduce challenges for real-time cognitive workload estimation in practical, dynamic environments. Therefore, there remains a need for lightweight, interpretable models using standard EEG configurations that can maintain predictive accuracy while reducing computational burden. A comprehensive summary of previous work on EEG-based CW estimation, including their methods and observed limitations, is presented in Table 1.

**Table 1:** Summary of literature on cognitive workload evaluation, key findings, and limitations.

Author(s)	Focus Area	Methodology	Key Findings	Limitations	Ref
Aricò et al., 2016	Passive brain-computer interfaces (BCIs) for mental state classification	Explored passive BCIs using EEG features for classification	Achieved ~ 80% accuracy in mental state classification	Task-specific BCIs; are not robust for generalized cognitive workload evaluation	^41^
Borghini et al., 2017	Classification of EEG features for cognitive workload and mental state analysis	Applied SVM and KNN for feature classification	Achieved ~ 85% accuracy in classification tasks	Limited dataset diversity; did not address dynamic network flexibility	^49^
Hashimoto et al., 2018	Machine learning in surgical performance analysis	Applied ML models for regression analysis of surgical performance data	Achieved R^2^ = 0.89; demonstrated the feasibility of ML in surgical performance analysis	Did not include cognitive workload evaluation or eye-tracking data	^45^
Salaken et al., 2020	Cognitive workload prediction using machine learning	Applied SVM and RF models for workload classification	Achieved ~ 88% accuracy in workload classification	Limited exploration of feature engineering and dataset size	^43^
Zhang et al., 2020	EEG classification using random forest	Focused on feature extraction and classification using RF models	Achieved ~ 92% classification accuracy	Did not integrate time–frequency domain features like PSD	^15^
Lin et al., 2021	Temporal EEG feature analysis for workload prediction	Implemented LSTM and deep learning models for time-series EEG data	Achieved ~ 88% accuracy in workload classification	Requires large datasets and high computational power	^46^
Shadpour et al., 2023	Surgical performance evaluation using EEG data and machine learning	Used GLMM-LASSO and linear models for regression analysis	Achieved R^2^ = 0.97; highlighted the importance of EEG data for performance evaluation	High computational demands; limited real-time applicability; reliance on high-density EEG data	^2^

Despite notable advancements in EEG-based CW assessment, many existing methods face limitations that hinder their practical deployment, particularly in real-time applications. Several studies, such as Shadpour et al.^2,]^^50,51^, rely on multi-channel or high-density EEG systems combined with complex feature extraction pipelines, including functional brain network analyses, temporal dynamics, and integration metrics^52–]^^54^. While these methods provide deeper insights into brain connectivity, they introduce substantial computational overhead, limiting the feasibility of real-time implementation.

Similarly, deep learning approaches like those proposed by Lin et al.^46^ have demonstrated strong predictive capabilities, but their reliance on large datasets and intensive training requirements makes them unsuitable for resource-constrained environments. In addition, many prior methods utilize intricate feature sets that require extensive preprocessing and domain-specific expertise, further complicating practical adoption. Although models such as GLMM and LASSO have achieved moderate to high accuracy, their computational demands and dependence on high-density EEG data continue to pose scalability challenges. Moreover, these gaps highlight the need for a more efficient, interpretable, and accessible approach that reduces computational complexity while maintaining or improving predictive performance.

## Methodology

**Fig. 2:**
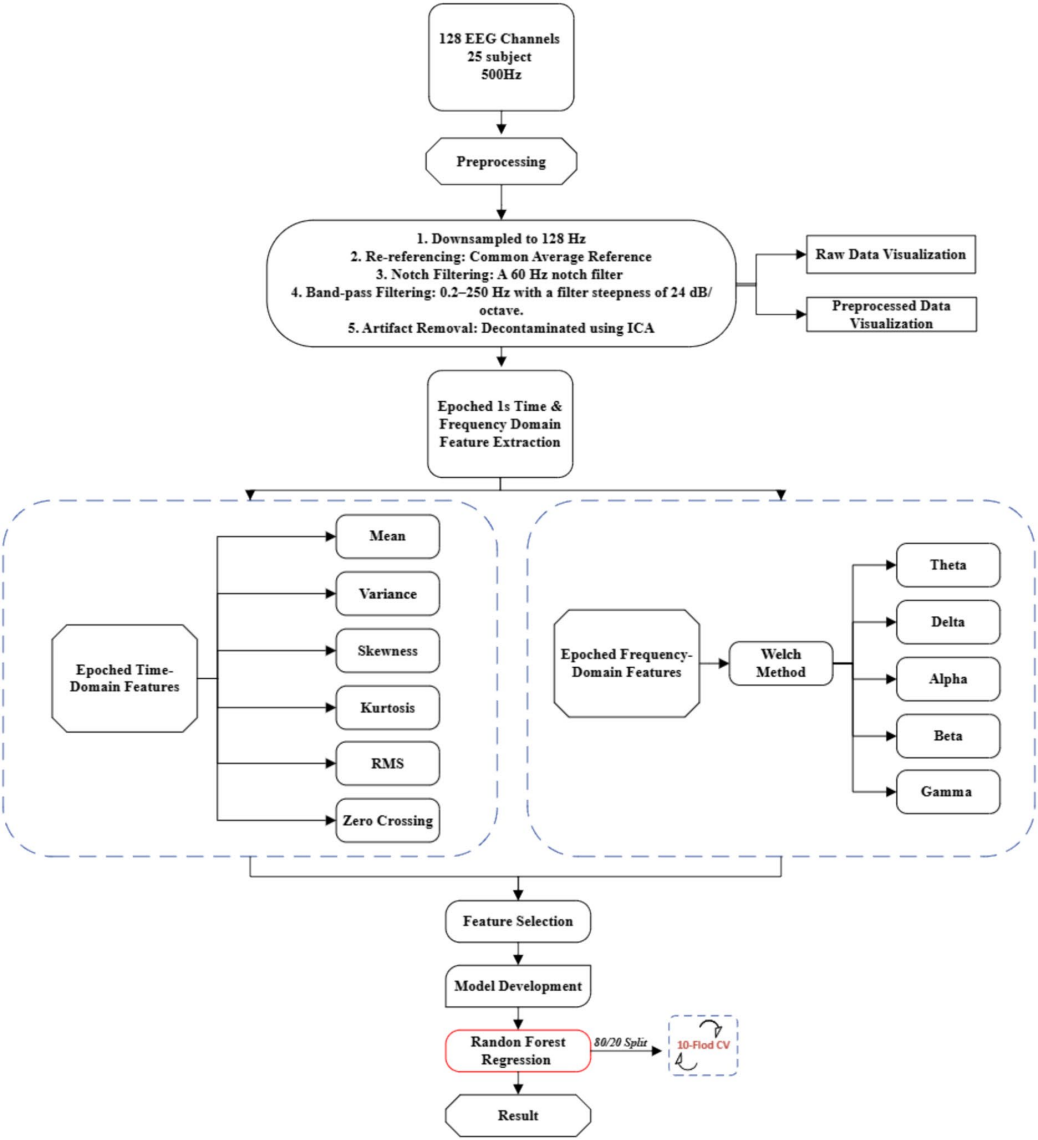
EEG data processing & machine learning for cognitive workload analysis.

The workflow for systematically analyzing EEG data and predicting cognitive workload is illustrated in Fig. 2. The initial steps involve recording and preprocessing the EEG signals, which include digital filtering, downsampling to 128 Hz, re-referencing using the Common Average Reference (CAR) method, artifact removal, and segmentation into epochs for analysis. After preprocessing, features are extracted from both the time and frequency domains. Time-domain features include mean, variance, skewness, kurtosis, RMS, and zero-crossing rate. Frequency-domain features are obtained using Welch’s method, which computes the power spectral density across canonical EEG frequency bands: delta, theta, alpha, beta, and gamma. Following feature extraction, selection techniques are applied to retain the most relevant predictors, which are then used to develop a machine learning model capable of mapping EEG features to varying levels of cognitive workload. After development and training, the models are evaluated using a systematic testing procedure (e.g., k-fold cross-validation) to assess their predictive performance using metrics such as R^2^, RMSE, and MAE. This integrated pipeline combines established EEG analysis methods with predictive modeling to enable robust and interpretable cognitive workload estimation.

The study proposed in Fig. 2 also follows structured methodology for estimating CW utilizing EEG data and machine learning techniques, with assured computational efficiency. The process initiates from data collection of a total of 25 participants, using 128-channel surface EEG at 500 Hz sampling rate, followed by a string of preprocessing steps. The data downsampled to 128 Hz findings ensured adequate signal processing, re-referencing with the Common Average Reference (CAR) method to enhance clarity of signals, placement of a notch filter at 60 Hz for the removal of power line noise, and band-pass filtering is adopted to accept signals from 0.2–250 Hz. Independent Component Analysis (ICA) is utilized for removing artifacts, thus ensuring that non-brain signals such as eye blinks do not interfere in the analysis. In this way, EEG signals both raw and preprocessed were visualized for quality inspection before feature extraction.

At this step of the analysis, the data would first be segregated into epochs of EEG recordings, followed by extraction of features both time-domain and frequency-domain features. The time-domain features comprise statistical metrics such as mean, variance, skewness, kurtosis, root mean square, and zero crossing. These features provide insight into variations and structure of EEG signals. Frequency-domain features are generated using Welch’s method. It analyzes data in the delta (0.5–4 Hz), theta (4–7 Hz), alpha (8–12 Hz), beta (13–30 Hz), and gamma (30–50 Hz) frequency bands, correlated in establishing cognitive states. Further refinement was applied to the features to retain only the most relevant predictors to reduce computational complexity with the remaining preserved predictive power.

RFR was selected for model development based on its capability to handle complex data structures and at the same time show computational efficiency compared to deep learning-based approaches. Unlike the previously proposed Gaussian mixture models^2^, which required an exceedingly high number of computations in drawing a GLMM and LASSO regression, this study offers a more accessible and scalable technique. The present work achieves an established benchmark for CW assessment with time-domain and frequency-domain features combined with an optimized feature selection and an efficient machine learning model, thus pointing out the feasibility of real-time implementation making this approach very suitable for practical applications in robotic-assisted surgery and other high-stakes settings where efficient cognitive workload monitoring is critical.

### Data acquisition and data preparation

The dataset used in this study, Electroencephalogram and Eye-Gaze Datasets for Robot-Assisted Surgery Performance Evaluation, was sourced from the PhysioNet platform (https://physionet.org/content/eeg-eye-gaze-data/1.0.0/)^2,55^. It consists of EEG recordings from 25 participants (8 females, mean age = 27.0 ± 5.93 years; 17 males, mean age = 39.53 ± 12.42 years) with varying levels of experience in robotic-assisted surgery (RAS). Task performance was conducted in an eyes-open condition. EEG signals were recorded at 500 Hz and later downsampled to 128 Hz. Each task block lasted approximately 5 min. Signals were segmented into 1-s epochs with 50% overlap, allowing a 2 Hz update frequency for feature extraction and model inference. The dataset was downloaded as CSV files containing time-series data from multiple subjects, with detailed information from various scalp EEG channel sensors. The raw data were examined and visualized to explore the overall characteristics of the signals and to identify possible noise or artifacts. Brain activity was recorded using a 128-channel surface EEG headset at a constant sampling rate of 500 Hz. Four EEG leads intended for electrooculogram (EOG) were excluded from the analysis to focus solely on brain activity. The study included twenty-five participants, aged between twenty and sixty-seven years, with varying levels of experience in robot-assisted surgery (RAS): Twelve participants had no prior exposure to RAS, four had less than a hundred hours of experience, and the remaining nine were highly experienced but with diverse proficiency levels.

**Fig. 3:**
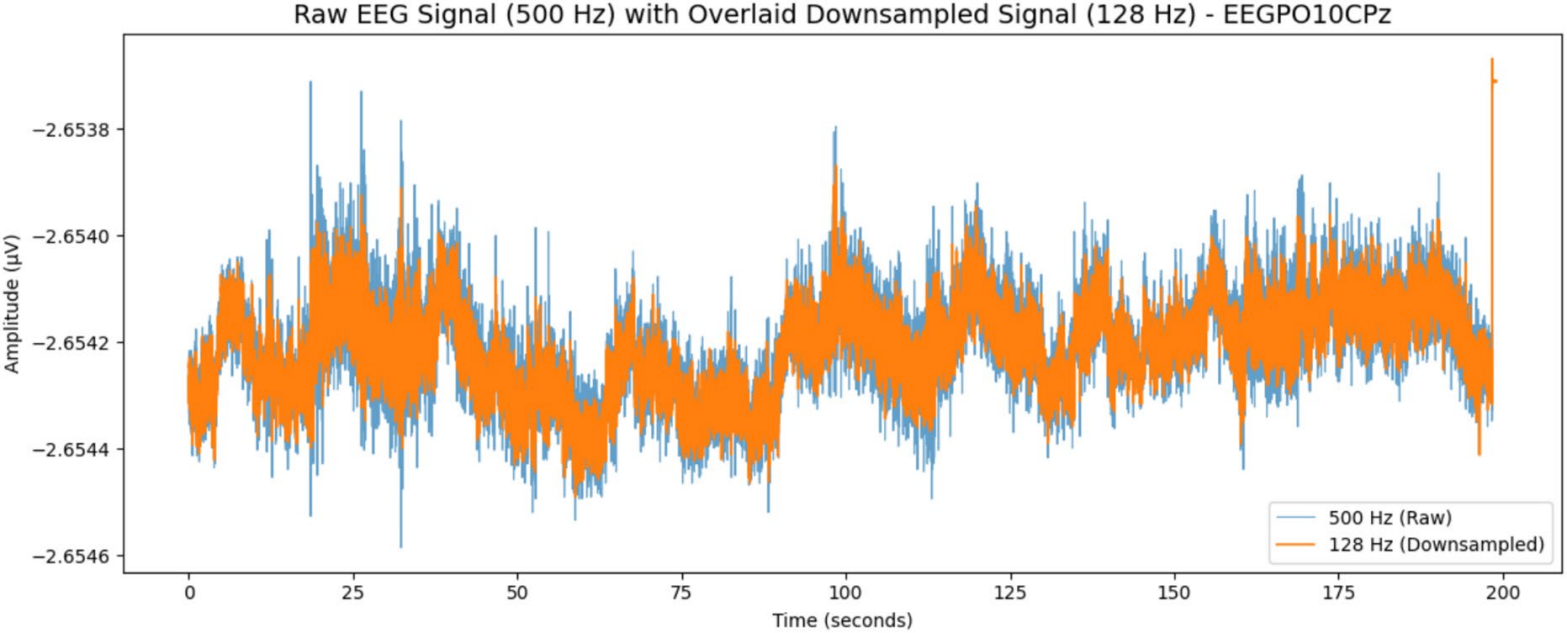
Comparison of EEG data before and after downsampling. The original 500 Hz signal (background) is overlaid with its 128 Hz downsampled version. Key signal features and overall trends remain consistent, with minimal loss of detail despite the reduced sampling rate.

As the first step of the data processing pipeline, the signals were assessed visually to understand their features and identify noise or artifacts likely to interfere with analysis accuracy. This stage is highly important, as raw EEG data often present considerable levels of noise, such as electrical interference, muscle distortions, or eye movement artifacts. Such anomalies may have the effect of misrepresenting the underlying brain activity intended to be detected. The visual inspection of the data allowed for early detection of such artifacts, along with the possibility of taking remedial action to ensure the quality and reliability of the data before the analysis. At this stage, typical artifact detection could, for example, identify sporadic spikes or irregular oscillations in the signal, with either filtering or artifact rejection processes being employed. Figure 3 illustrates this procedure, showing the raw data before cleaning with all the inherent noise present on the signals.

As a preliminary step in the data processing pipeline, raw EEG signals originally recorded at 500 Hz were visually inspected to identify common sources of contamination such as muscle artifacts, eye blinks, and environmental electrical noise. This initial quality control ensured that the integrity of cognitive signal components was preserved before subsequent processing, raw EEG signals prior to cleaning and demonstrates the impact of downsampling on signal fidelity. To enhance computational efficiency and facilitate near-real-time analysis, the EEG signals were downsampled from 500 to 128 Hz following anti-aliasing filtering^56–61^. Downsampling significantly reduces data size and processing time while preserving the frequency components critical to cognitive workload analysis^62^. Specifically, the target frequency range of 0.5–50 Hz encompassing delta, theta, alpha, beta, and low gamma bands is well preserved at 128 Hz due to the Nyquist criterion, which allows accurate reconstruction of signals up to 64 Hz.

This practice is well-supported in EEG literature. For example, Li et al. applied downsampling to 128 Hz in emotion recognition tasks and demonstrated that model performance remained high while computation costs were reduced^16^. Similarly, Cooney et al.^63^ and Ding et al.^64^ used downsampling to 128 Hz for imagined speech classification and ERP-based speller systems, respectively, achieving reliable results without significant loss of signal information. Shadpour et al. also processed prefrontal EEG data sampled at 500 Hz but focused their analysis on the < 65 Hz band, effectively demonstrating that a higher acquisition rate is not essential for cognitive state estimation tasks^2,65–68^. To validate this preprocessing step, visual comparisons were made between EEG traces before and after downsampling. Figure 3 shows that key temporal patterns and frequency signatures remain intact, confirming that the downsampled data preserved essential signal features required for robust cognitive workload modeling. This balance between preserving physiological relevance and achieving computational efficiency is especially important in high-stakes contexts such as real-time monitoring during robot-assisted surgery, where analytical speed and data fidelity are both critical^3,69–73^.

### Preprocessing

**Fig. 4:**
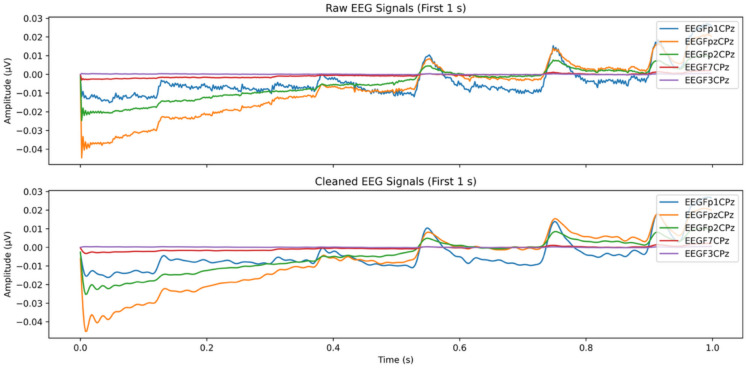
Visual representation of the preprocessing steps applied to EEG data. Example shown from a single Cz electrode trace, illustrating raw EEG before preprocessing and cleaned EEG after preprocessing for cognitive workload analysis.

This study involved preprocessing pipeline for EEG data stored in EDF files using the Python programming environment. The pipeline automates the extraction and cleaning of EEG signals to ensure data quality for subsequent analysis. EEG signals were recorded using a DC-coupled amplifier system (ANT Neuro)^2^. All EDF files were loaded using the MNE library, and channel names were extracted for labelling at the outset. During acquisition, the reference electrode was placed at Cz (central vertex), and the ground electrode was located at AFz^2^. This referencing scheme is commonly used in high-density EEG research to reduce global noise and improve spatial resolution and series of preprocessing steps were adopted: (1) Re-referenced the signals to the average potential across all channels by employing the Common Average Referencing algorithm to minimize noise. In this approach, the mean potential is calculated at each time point and subtracted from each channel, which attenuates spatially correlated noise and common-mode interference (e.g., environmental electrical noise, reference drift) while preserving localized neural activity. (2) A notch filter at 60 Hz followed by a band-pass filter (0.2–250 Hz) to reduce irrelevant low- and high-frequency noise this artifact correction, using the ANT Neuro ASA framework. As part of this pipeline, a band-pass filter ranging from 0.2 to 250 Hz (24 dB/octave) was applied to facilitate robust artifact detection and cleaning. This broader filter range enabled the capture of low-frequency drift and high-frequency muscle artifacts, both of which were addressed during source separation. Importantly, this filter was not used to define EEG sub-band features^2^. (3) ICA for artifacts related to ocular movements (e.g., eye blinks) and muscle activity were removed using Independent Component Analysis (ICA), a robust signal decomposition technique widely employed in EEG preprocessing. The ICA-based artifact correction was performed through a hybrid approach either automatically or with manual component selection depending on the nature and clarity of the artifacts. This pipeline was followed by visual inspection to confirm the rejection of non-neural components. Approximately 10–15% of the EEG epochs were excluded due to residual noise contamination or irrecoverable artifacts. The combined use of ICA and manual verification ensured the integrity of the remaining data. This dual-layered preprocessing strategy supports reproducibility and scalability, which are essential for handling large EEG datasets in statistical and machine learning applications. The impact of preprocessing was validated through visual comparison of the EEG signal before and after artifact removal, as demonstrated in Fig. 4.

In EEG signal analysis, spectral variations were examined using the Short-Time Fourier Transform (STFT) to track frequency content over time. A one-second Kaiser moving window with a 50% overlap was employed to maintain a balance between time and frequency resolution while minimizing spectral leakage. Subsequently, the PSD was computed from the resulting spectrogram using Welch’s method. The PSD provides power distribution across key EEG frequency bands, including theta (4–8 Hz), alpha (8–12 Hz), beta (13–30 Hz), and gamma (30–50 Hz), which are closely associated with critical cognitive functions and brain activity and elevated broadband noise and low-frequency drifts, whereas the cleaned spectral clarity with attenuated noise and sharper band delineation, facilitating more accurate cognitive workload assessment.

### EEG channels and mapping

**Fig. 5:**
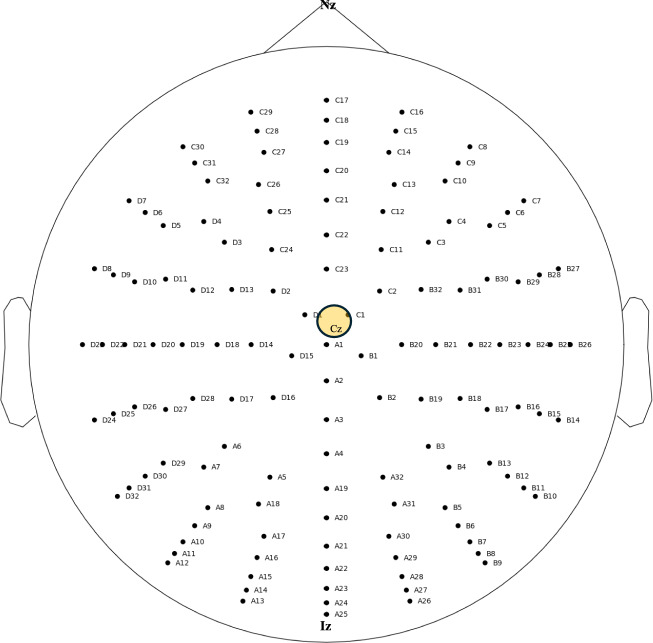
Electrode placement used for data collection of 128-channel surface EEG system.

Each EEG file contained data recorded from a 128-channel surface EEG headset. In certain recordings, specific channels including F8, POz, AF4, AF8, F6, and FC3 did not capture high-quality signals. Metadata such as channel labels, channel numbers, and recording time (in seconds) was stored within the EDF structure of every EEG file. Signals labelled as "EEGHEOGRCPz," "EEGHEOGLCPz," "EEGVEOGUCPz," and “EEGVEOGLCPz” originated from electrooculography (EOG) recordings and were excluded from the EEG analysis. Additionally, some EEG recordings could not be completed due to technical issues. Figure 5 provides an overview of the 128-channel surface EEG electrode placement. During acquisition, the reference electrode was placed at Cz (central vertex) and the ground electrode at AFz^2^.

### Spatiotemporal EEG analysis

**Fig. 6:**
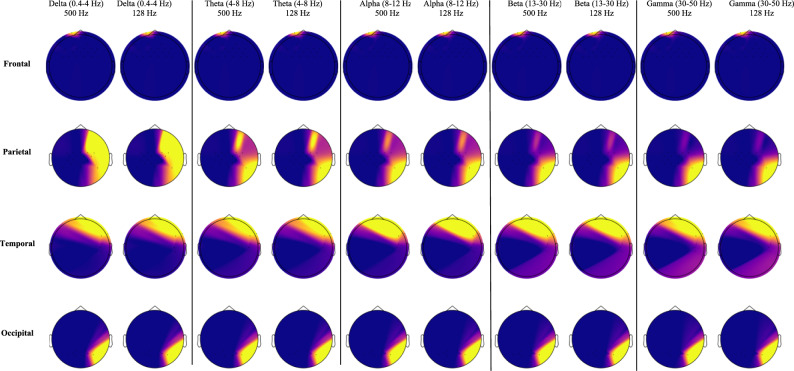
Topographic maps of EEG power across delta-gamma bands for frontal, parietal, temporal, and occipital regions at 500 Hz (left) and 128 Hz (right). Despite reduced sampling, key spatial patterns such as elevated parietal, temporal beta-gamma activity associated with sensory integration and working memory are preserved, supporting robust low-rate feature extraction.

To characterize the spatial and spectral structure of EEG signals, both band-specific power topographic maps (topoplots) and time–frequency spectrograms were generated, as shown in Fig. 6. EEG topoplots were computed across canonical frequency bands delta (0.5–4 Hz), theta (4–8 Hz), alpha (8–13 Hz), beta (13–30 Hz), and gamma (30–50 Hz) to visualize the spatial distribution of oscillatory activity across the scalp. Comparisons between recordings sampled at 500 Hz and those downsampled to 128 Hz confirmed that topographical integrity was preserved across frequencies, ensuring consistency in cortical activity localization. These scalp maps validated the anatomical stratification of brain regions (frontal, parietal, temporal, occipital) used for region-wise model training.

EEG power topoplots as shown Fig. 6 is obtained from the original 500 Hz recordings and downsampled 128 Hz signals. For each participant and condition, the mean PSD was calculated at each electrode using Welch’s method, which segments the signal into overlapping windows, applies a Hanning window to reduce spectral leakage, and averages the squared magnitudes of the resulting Short-Time Fourier Transform (STFT) to obtain a robust estimate of spectral power. The computed PSD values were then spatially mapped to the standardized 2-D scalp layout of the 128-channel EEG montage using spherical spline interpolation^74^. This interpolation produces smooth gradients between electrode positions, generating continuous color-coded maps in which warmer colors indicate higher relative power and cooler colors indicate lower power.

Consistent with prior EEG research^75,76^ the downsampled maps retained the principal topographical patterns observed at 500 Hz, confirming that key spatial features are preserved even with reduced sampling rates. This is critical for real-time or resource-constrained applications, as it supports the feasibility of low-rate acquisition without compromising neurophysiological interpretability^2,77,78^. In our study, these maps reveal region-specific spectral signatures associated with cognitive workload modulation or example, elevated beta and gamma power in the parietal and temporal cortices during high-demand segments, consistent with patterns associated with enhanced sensory integration, attentional control, and working memory load in previous studies^74,78,79^. These spatial distributions also validate the anatomical segmentation used for region-wise model training, ensuring that the frequency-domain features provided to the Random Forest regression framework are both statistically predictive and neuro-physiologically grounded.

**Fig. 7:**
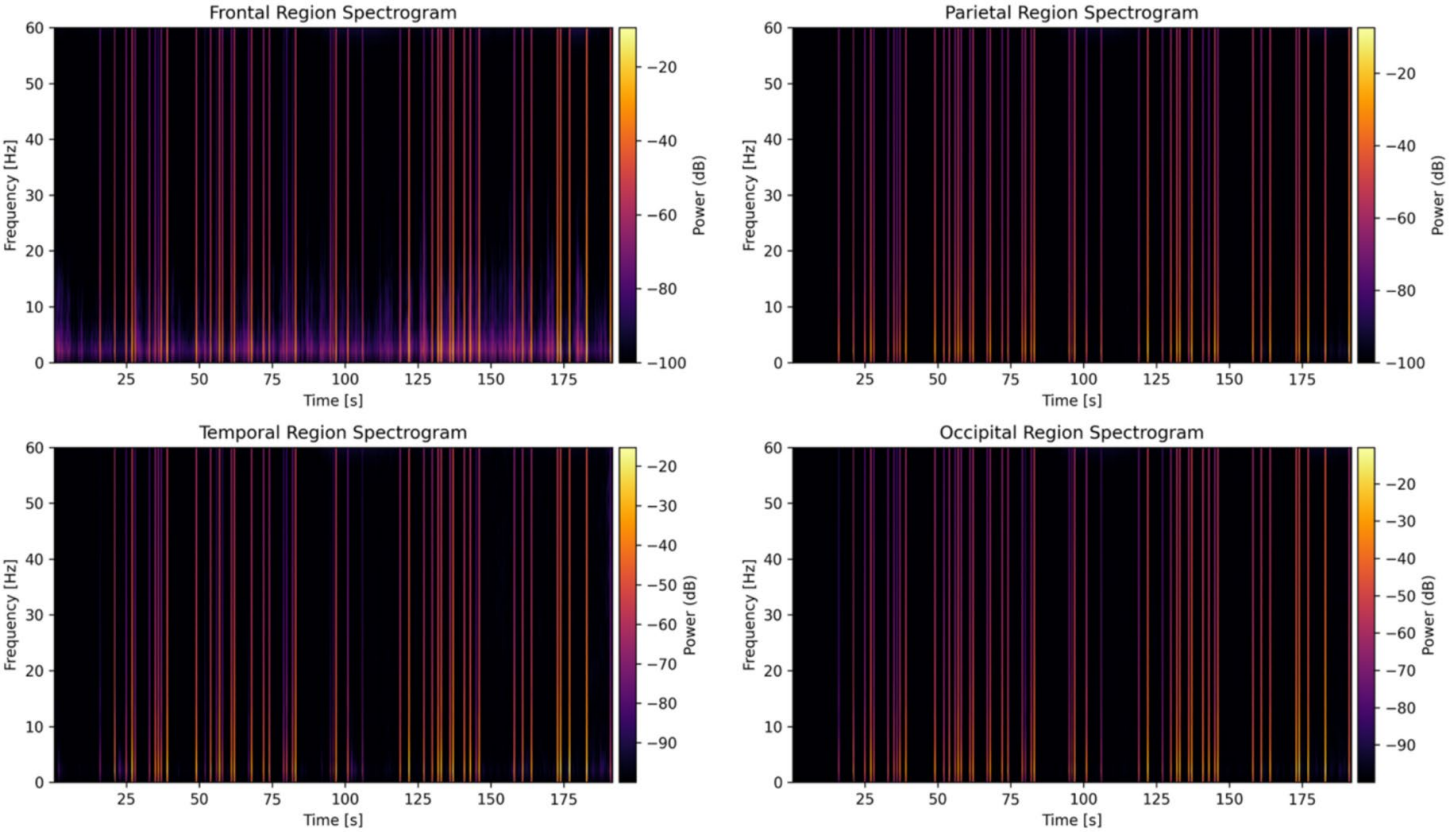
Time–frequency spectrograms of EEG signals sampled at 500 Hz for four cortical regions: Frontal (Fz), Parietal, Temporal, and Occipital. Power spectral densities were computed using short-time Fourier transform (STFT) with a 256-sample window and 50% overlap. These plots illustrate temporal evolution of oscillatory power across canonical EEG bands, highlighting distinct spectral dynamics associated with cognitive workload processing.

**Fig. 8:**
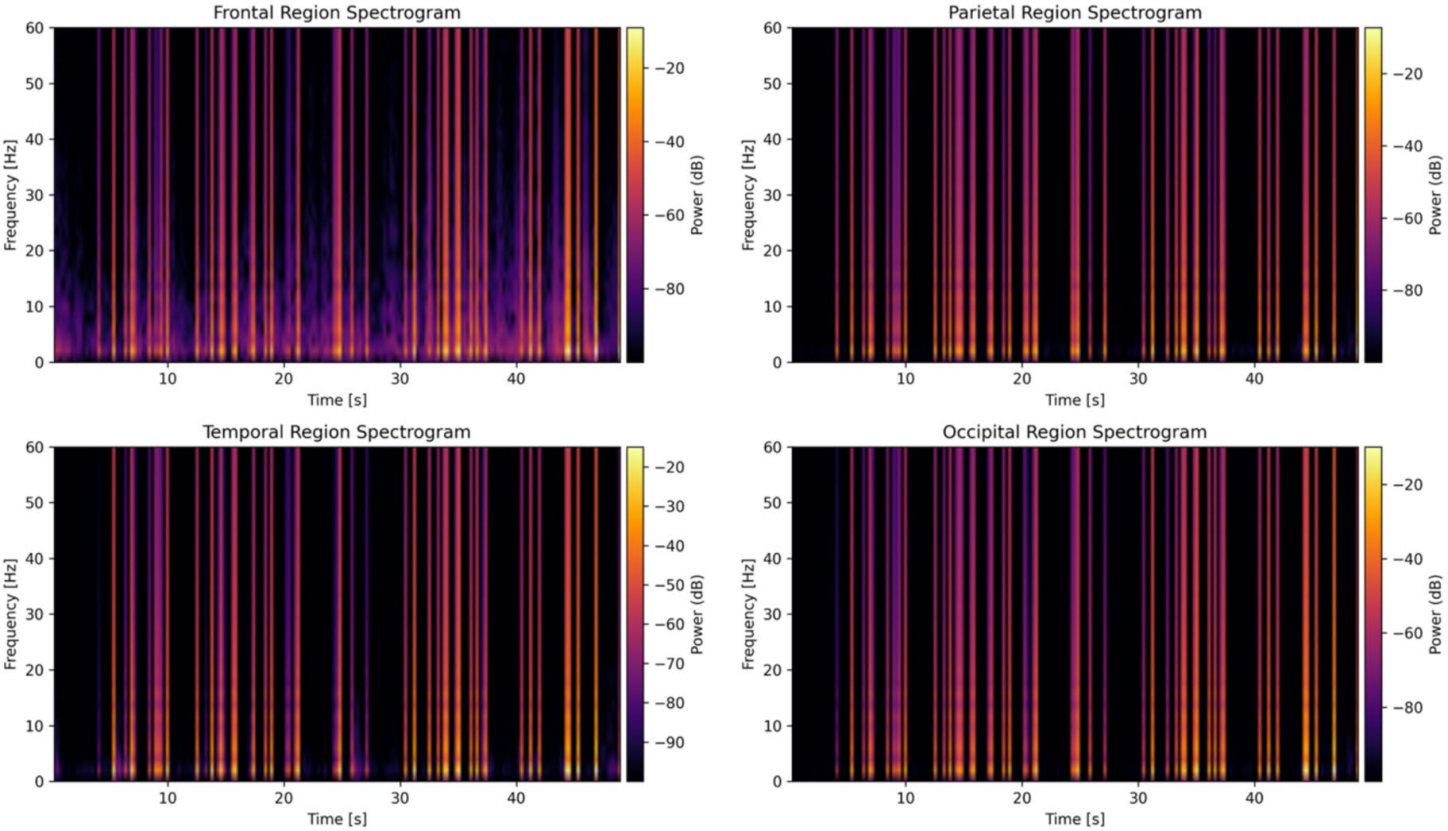
Time–frequency spectrograms of EEG signals downsampled to 128 Hz for the same four cortical regions. While temporal resolution is reduced relative to 500 Hz, the spectrograms retain key low-frequency features (e.g., delta, theta), validating the feasibility of lower-rate acquisition for real-time cognitive workload monitoring.

To capture spatiotemporal EEG dynamics, time–frequency spectrograms were computed from one subject’s data, as shown in Figs. 7 and 8 using short-time Fourier transforms (STFT) on representative channels from each cortical region. The STFT parameters were identical in both cases (nperseg = 256 samples, 50% overlap), but because nperseg (number of samples per segment) is specified in samples, the effective window length depends on sampling rate 0.512 s at 500 Hz versus 2.0 s at 128 Hz altering the time–frequency trade-off and slightly changing the visual appearance of transient events, as seen in Fig. 8. These spectrograms illustrate the evolution of spectral power across time and frequency, offering a dynamic perspective on neural activation. Consistent with prior findings, increased frontal theta activity a known correlate of heightened cognitive workload was observed during high-demand segments. A meta-analysis reported a significant association between frontal theta power and cognitive workload, underscoring the relevance of these spectral patterns^78^. These visual analyses support the biological plausibility of the extracted features and validate the choice of frequency-domain predictors used in the regression modeling framework. All recordings were visually inspected to remove ocular and muscle artifacts prior to analysis.

### Feature extraction

In this study, EEG signal analysis was carried out by segmenting continuous recordings into fixed-duration epochs to enable structured temporal analysis. Each epoch was set to a length of 1 s, and data were sampled at a rate of 128 Hz, resulting in 128 samples per epoch. This window size was selected to ensure adequate frequency resolution while maintaining compatibility with real-time system constraints. Epoch segmentation facilitates localized feature extraction, allowing the capture of transient and stable neural patterns related to cognitive workload. From each 1-s epoch, both time-domain and frequency-domain features were extracted to provide a multidimensional representation of the underlying brain activity. Time-domain features included statistical descriptors such as the mean, variance, skewness, and kurtosis, as well as energy-related metrics such as the root mean square (RMS) and zero-crossing rate, comprehensive list of the extracted time-domain and frequency-domain features, including their definitions and formulas is summarized in Table 2. These features collectively describe the amplitude distribution, waveform symmetry, and fluctuation characteristics of the EEG signal within a given epoch^80,81^.

**Table 2:** EEG signal features, categorizing time-domain and frequency-domain metrics with descriptions, formulas, and references.

**Feature**	**Category**	**Description**	**Formula**	**Reference**
Mean	Time-Domain	Average amplitude of the signal; indicates general brain activity	$\upmu =\frac{1}{\mathrm{N}}{\sum }_{\mathrm{i}=1}^{\mathrm{N}}{\mathrm{x}}_{\mathrm{i}}$ μ = Mean value of the signal x_i_ = EEG signal amplitude at sample i N = Total number of samples	^80^
Variance	Time-Domain	Variability of signal values; reflects cognitive activity or workload	${\upsigma }^{2}=\frac{1}{\mathrm{N}}{\sum }_{\mathrm{i}=1}^{\mathrm{N}}{\left({\mathrm{x}}_{\mathrm{i}}-\upmu \right)}^{2}$ σ^2^ = Variance	^80^
Skewness	Time-Domain	Asymmetry of signal distribution; indicates abnormal brain activity	${\mathrm{Skewness}}=\frac{\frac{1}{\mathrm{N}}{\sum }_{\mathrm{i}=1}^{\mathrm{N}}{\left({\mathrm{x}}_{\mathrm{i}}-\upmu \right)}^{3}}{{\left(\frac{1}{\mathrm{N}}{\sum }_{\mathrm{i}=1}^{\mathrm{N}}{\left({\mathrm{x}}_{\mathrm{i}}-\upmu \right)}^{2}\right)}^{3/2}}$	^80^
Kurtosis	Time-Domain	Peakedness or flatness of signal distribution; identifies signal spikes	${\mathrm{Kurtosis}}=\frac{\frac{1}{\mathrm{N}}{\sum }_{\mathrm{i}=1}^{\mathrm{N}}{\left({\mathrm{x}}_{\mathrm{i}}-\upmu \right)}^{4}}{{\left(\frac{1}{\mathrm{N}}{\sum }_{\mathrm{i}=1}^{\mathrm{N}}{\left({\mathrm{x}}_{\mathrm{i}}-\upmu \right)}^{2}\right)}^{2}}$	^80^
Root Mean Square (RMS)	Time-Domain	Energy or magnitude of the signal; higher values indicate increased brain activity	$\mathrm{RMS}=\sqrt{\frac{1}{\mathrm{N}}{\sum }_{\mathrm{i}=1}^{\mathrm{N}}{\mathrm{x}}_{\mathrm{i}}^{2}}$	^80^
Zero Crossing	Time-Domain	Number of signal oscillations; high crossings indicate increased neural activity	${\mathrm{ZC}}={\sum }_{\mathrm{i}=1}^{\mathrm{N}-1}1\left({\mathrm{x}}_{\mathrm{i}}\cdot {\mathrm{x}}_{\mathrm{i}+1}<0\right)$ x_i_ = EEG signal amplitude at sample i x_i_ + 1 = EEG signal amplitude at the next sample $\sum 1$= Indicator function**,** which outputs: **1** if x_i_ ⋅ x_i_ + 1 < 0 (i.e., there is a zero crossing). **0** otherwise − N = Total number of samples	^80^
Delta Band Power	Frequency-Domain	Power in 0.5–4 Hz; reflects relaxation or mental fatigue	${\mathrm{P}}_{\updelta }={\sum }_{\mathrm{f}=0.5}^{4}\mathrm{PSD}\left(\mathrm{f}\right)$ -P_δ_ = Power in delta band - PSD(f) = Power Spectral Density at frequency f	^80^
Theta Band Power	Frequency-Domain	Power in 4–8 Hz; associated with memory encoding and problem-solving	$P_{\theta } = \sum\limits_{f = 4}^{8} {PSD(f)}$ -P_θ_ = Power in theta band	^80^
Alpha Band Power	Frequency-Domain	Power in 8–13 Hz; reflects relaxation and focus; reduced power indicates cognitive load	$P_{\alpha } = \sum\limits_{f = 8}^{13} {PSD(f)}$ -Pα = Power in alpha band	^80^
Beta Band Power	Frequency-Domain	Power in 13–30 Hz; associated with active thinking and decision-making	${\mathrm{P}}_{\upbeta }={\sum }_{\mathrm{f}=13}^{30}\mathrm{PSD}\left(\mathrm{f}\right)$ -P_β_ = Power in beta band	^80^
Gamma Band Power	Frequency-Domain	Power in 30–50 Hz; indicates high-level cognitive processes such as multitasking or solving complex problems	${P}_{\upgamma }={\sum }_{f=30}^{50}PSD\left(f\right)$ -Pγ = Power in gamma band	^80^

For frequency-domain analysis, PSD was estimated using Welch’s method, a well-established, non-parametric spectral estimation technique. Welch’s method involves segmenting each epoch into overlapping sub-windows (typically with 50% overlap), applying a Hanning window to reduce spectral leakage, computing the Short Fast Fourier Transform (SFFT) for each sub-window, and averaging the squared magnitudes of the resulting periodograms. This results in a smoothed PSD estimate that improves the signal-to-noise ratio and enhances spectral resolution. Unlike filter-based approaches such as Finite Impulse Response (FIR) filtering or wavelet decomposition, Welch’s method does not impose phase distortions or require explicit sub-band isolation, making it computationally efficient and highly suitable for applications that require real-time performance with spectral fidelity.

The PSD obtained via Welch’s method was then integrated over predefined frequency intervals corresponding to canonical EEG sub-bands: Delta (0.5–4 Hz), Theta (4–8 Hz), Alpha (8–13 Hz), Beta (13–30 Hz), and Gamma (30–50 Hz). These bands are widely studied and are known to reflect diverse cognitive and physiological states. For instance, delta and theta bands are associated with drowsiness and cognitive processing, alpha is linked to relaxation and inhibition, beta with active attention, and gamma with high-level cognitive functions. Each EEG channel was processed independently across all epochs, and metadata such as channel identity, file origin, and epoch index were appended to the feature matrix. The complete feature set was consolidated into a structured CSV file to enable seamless integration with downstream statistical modeling and machine learning pipelines.

To ensure robustness, the preprocessing and extraction pipeline incorporated systematic file validation, fault tolerance, and automatic logging of errors. These mechanisms were implemented to handle corrupt files, missing data, or structural inconsistencies, thereby ensuring that the analysis pipeline could scale across large datasets with minimal human intervention. This dual-domain feature extraction strategy integrating temporal signal shape with frequency-specific spectral power provides a comprehensive and high-resolution depiction of neural activity, aligning with standard practices in cognitive EEG research. It is particularly well-suited for cognitive workload modeling, as demonstrated in prior work^2,82^, and supports near real-time applications in neuro-ergonomics and surgical performance monitoring^83^.

### Feature importance

Feature selection was conducted using the embedded feature importance scores provided by the RFR. This approach ranks input features by their contribution to the model’s performance, measured via the mean decrease in impurity (MDI) across all decision trees in the ensemble. As a non-parametric method, Random Forest is well-suited for EEG analysis due to its robustness to noise, ability to capture nonlinear feature interactions, and efficiency in high-dimensional datasets. Using this model, this study identified the most influential features for predicting workload-associated cortical activity across the frontal, temporal, parietal, and occipital regions.

Feature importance analysis elucidated that Root Mean Square (RMS), Kurtosis, Skewness, and PSD features in classical EEG bands were among the most predictive metrics. RMS is a critical measure of signal energy and amplitude stability and was found to be particularly informative for frontal cortex activity, which is known to reflect executive functions and decision-making processes. Kurtosis, representing peakedness or burst activity, proved useful in identifying transient neural activations, especially in the temporal cortex, a region involved in memory and auditory processing. Skewness, quantifying signal asymmetry, offered insight into signal deviations from baseline and dynamic shifts in cognitive workload. Additionally, band-specific PSD features in delta, theta, alpha, beta, and gamma frequencies were strongly linked to functional brain states such as attention modulation, memory load, and higher-order cognitive processes. The multidimensional feature ranking supports the neurophysiological relevance of our EEG descriptors and validates the model’s ability to distinguish workload levels across cortical regions.

**Fig. 9:**
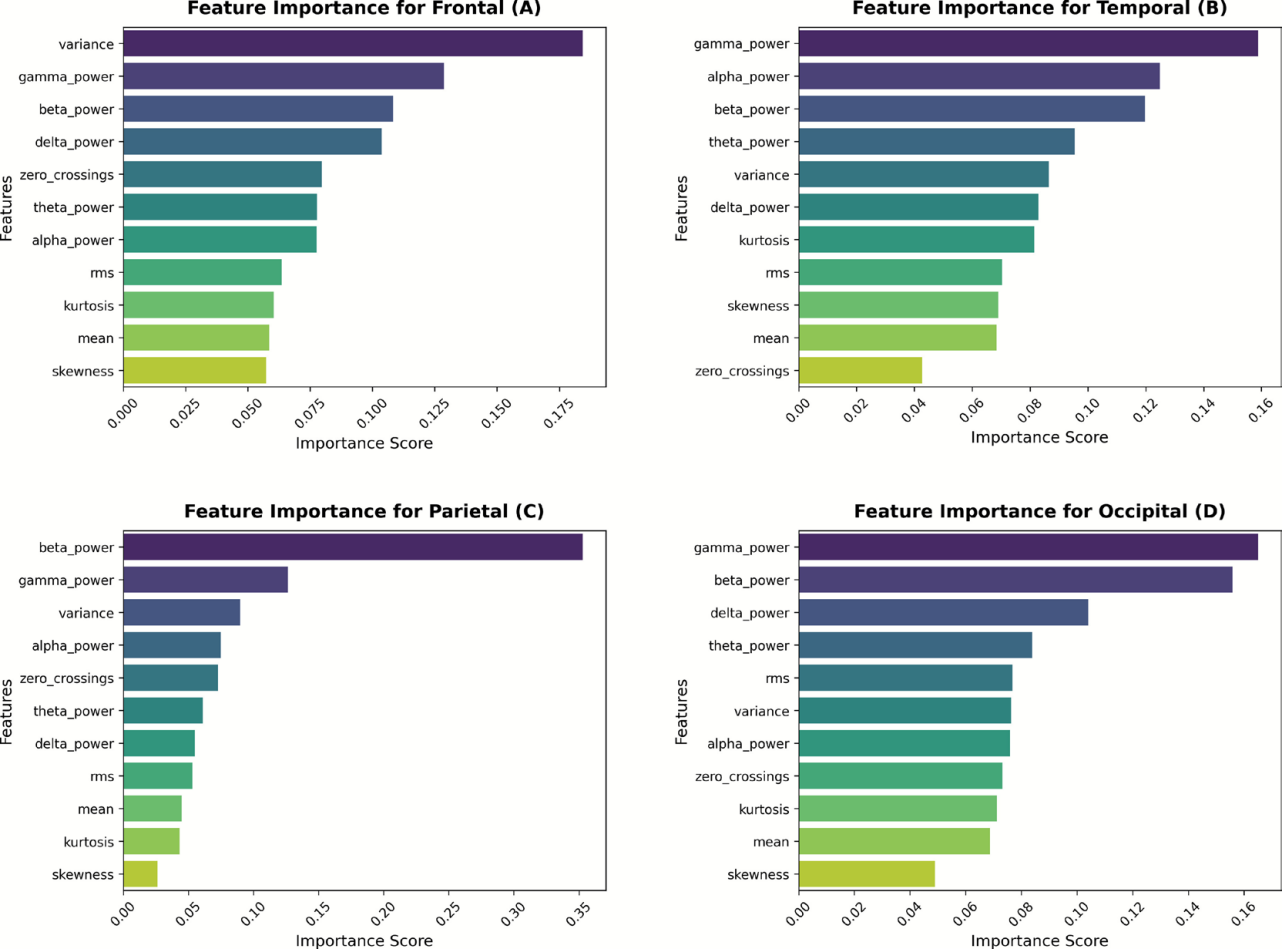
(a-d) Feature importance scores across different brain regions (**a**) Frontal, (**b**) Temporal, (**c**) Parietal, and (**d**) Occipital showing the dominant EEG features contributing to cognitive workload assessment.

As presented in Fig. 9 (a-d), feature importance differed across cortical regions, emphasizing specific region-based contributions to cognition. In the frontal cortex (Fig. 9 a), variance and gamma were shown to be the two most important features, reflecting their critical role in executive functions and decision-making. Likewise, in the temporal cortex (Fig. 9 b), associated with memory and auditory processing, gamma and alpha power were the prominent influences, strongly reaffirming previous observations on temporal lobe functions. In the parietal cortex (Fig. 9 c), responsible for sensory integration and spatial awareness, beta power and gamma power hold the greatest significance, supporting its role in processing multimodal sensory information. Finally, in the occipital cortex (Fig. 9 d), which governs visual processing, gamma power and beta power are the most dominant features, aligning with the region’s specialization in visual perception and analysis. These findings emphasize the region-specific relevance of EEG-derived features and their potential for enhancing neurophysiological modeling. By identifying the most influential features in cortical activity, this study contributes to a deeper understanding of cognitive workload assessment, brain-computer interface (BCI) development, and human–computer interaction, paving the way for more precise and interpretable neurophysiological models.

**Fig. 10:**
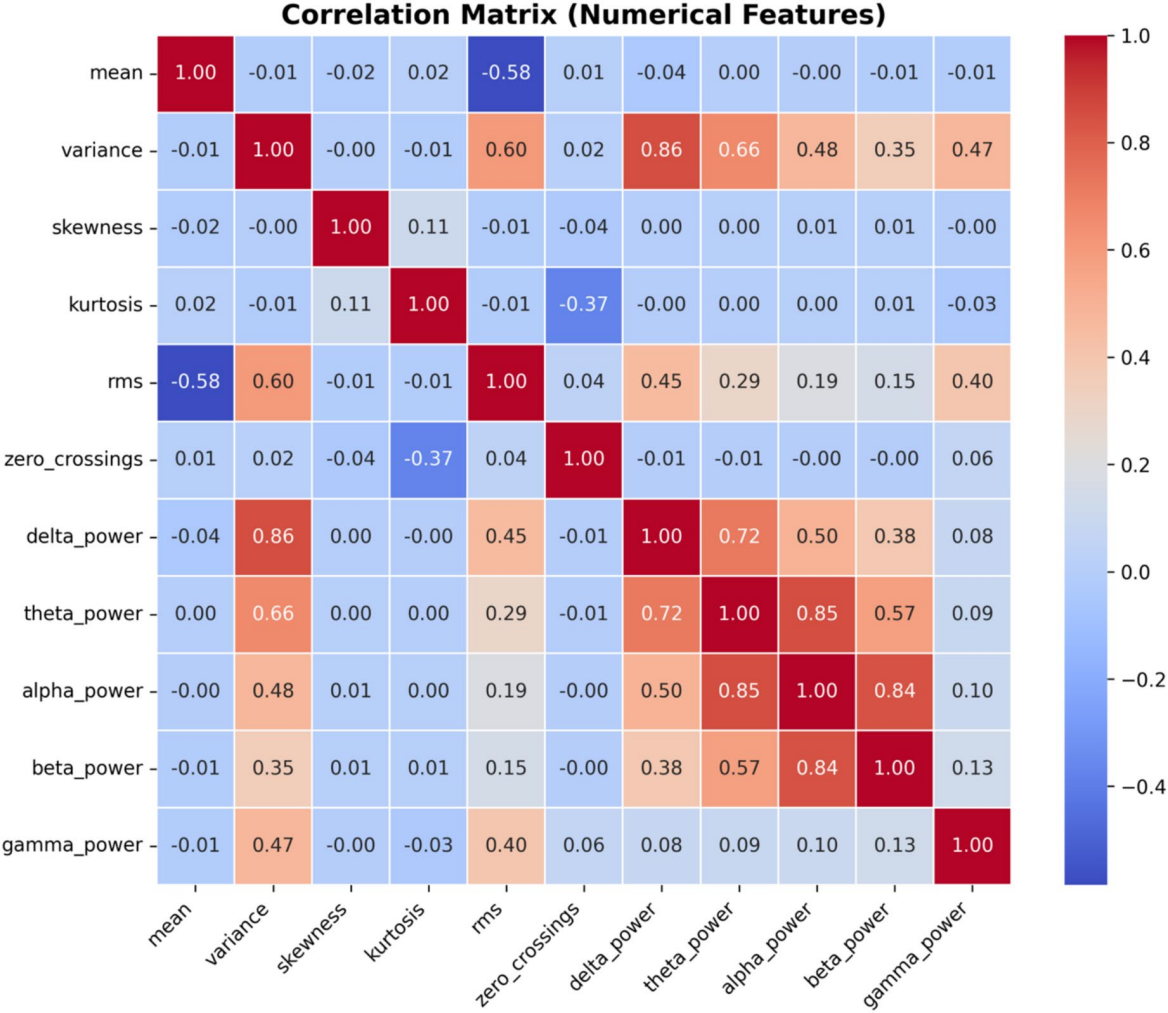
Correlation matrix showing relationships between EEG features, aiding feature selection for cognitive workload analysis.

The correlation matrix in Fig. 10 visualizes the relationships between numerical features, with colour intensity representing the strength and direction of correlation. Delta power shows a strong positive correlation with variance (0.86) and theta power (0.72), suggesting shared underlying patterns. In contrast, mean and RMS have a strong negative correlation (-0.58), indicating they capture opposing signal characteristics. Some features, like zero crossings, show weak or no correlation with others, highlighting their independent contribution. To find redundant features and choose the best predictors for cognitive workload modeling, this matrix is essential.

### Model development

The development of a robust machine learning pipeline for EEG-based cognitive workload estimation requires careful integration of physiologically meaningful features, appropriate algorithmic selection, and rigorous validation protocols. In this study, a supervised regression framework was employed to estimate region-specific cortical activation patterns associated with CW from quantitative EEG features extracted across frontal, temporal, parietal, and occipital regions. The target variable was defined as the mean EEG activation (per epoch) within each cortical region, calculated after artifact rejection and preprocessing. These activation values were treated as physiological proxies for cognitive workload, an approach supported by prior studies linking regional EEG amplitude and band power, particularly frontal theta and parietal beta, to workload modulation during complex tasks such as air traffic control, multitasking, and surgical simulation^84–86^. By framing the task as a multi-target regression problem, the model learned mappings between time-domain descriptors and frequency-domain spectral measures and the corresponding workload-associated cortical activation levels, enabling spatially resolved predictions without reliance on subjective workload scores.

Features used for modeling included time-domain statistics (mean, variance, skewness, kurtosis, RMS, zero-crossing rate) and frequency-domain measures obtained via PSD estimation using Welch’s method. PSDs were computed for canonical EEG bands delta (0.5–4 Hz), theta (4–8 Hz), alpha (8–13 Hz), beta (13–30 Hz), and gamma (30–50 Hz) which are widely reported to index changes in cognitive and affective states relevant to workload monitoring^76,84,85,87^. Each EEG epoch underwent preprocessing, including artifact removal, normalization, and imputation of missing values, and cortical region identifiers were one-hot encoded to support structured region-specific modeling. The dataset was initially partitioned using an intra-subject 80/20 train-test split stratified by cortical area to ensure balanced representation, and model training was augmented using tenfold cross-validation to minimize sampling bias and enhance generalizability.

For regression modeling, the RFR was selected as the primary algorithm due to its strong performance in high-dimensional, non-linear domains such as EEG signal analysis. RFR, an ensemble technique, constructs multiple decision trees using bootstrap aggregation and randomized feature selection, then aggregates their outputs, thus reducing model variance and mitigating overfitting. Additionally, RFR offers feature importance scores, which improve model interpretability, an asset in cognitive neuroscience contexts. For comparative analysis and benchmarking, additional models including SVR, Linear Regression, and XGBoost Regressor were also implemented. SVR is well-suited for modeling non-linear relationships with controlled complexity through kernel functions, while XGBoost, a gradient boosting algorithm, provides high efficiency and regularization mechanisms. These models were evaluated under identical preprocessing and feature extraction pipelines to ensure fair comparison. Overfitting was addressed through careful monitoring of training versus test performance, combined with statistical testing (e.g., paired t-tests) to assess the significance of observed differences (p < 0.0001). By integrating biologically motivated features, ensemble learning techniques, and rigorous validation strategies, this modeling framework lays the foundation for accurate, interpretable, and scalable EEG-based cognitive workload estimation applicable in high-stakes environments such as surgery and aviation.

## Result

The dataset underwent preprocessing steps including missing value imputation, feature normalization, and one-hot encoding of cortical regions to enable region-specific modeling. Data were partitioned into training (80%) and testing (20%) subsets for each cortical area using stratified sampling to preserve class balance. RFR was configured with 100 estimators, balancing predictive accuracy with computational efficiency. Model performance was evaluated using the coefficient of determination (R^2^), Mean Absolute Error (MAE), and Root Mean Square Error (RMSE). Across all cortical regions, R^2^ values exceeded 0.93, with the temporal cortex achieving the highest value (0.9951). MAE and RMSE values were consistently low across all regions, and p-values from significance testing were p < 0.0001 for all cases, indicating statistical reliability of the results (Table 3). For the frontal cortex, the model achieved an R^2^ of 0.9329 and RMSE of 0.0238. The temporal cortex achieved an R^2^ of 0.9947 and RMSE of 0.0019. In the parietal cortex, the model yielded an R^2^ of 0.9692 and RMSE of 0.0004, while the occipital cortex achieved an R^2^ of 0.9635 and RMSE of 0.0002. RFR model achieved high predictive accuracy and low error rates across all cortical regions.

**Table 3:** Performance evaluation of cognitive workload using random forest regression: model analysis across four cortical regions.

Brain Area	Number of Data Points	Accuracy (R^2^)	Root Mean Square Error (RMSE)
Frontal	263,221	0.9329	0.0238
Temporal	74,941	0.9947	0.0019
Parietal	111,675	0.9692	0.0004
Occipital	74,450	0.9635	0.0002

### Regression performance visualization

**Fig. 11:**
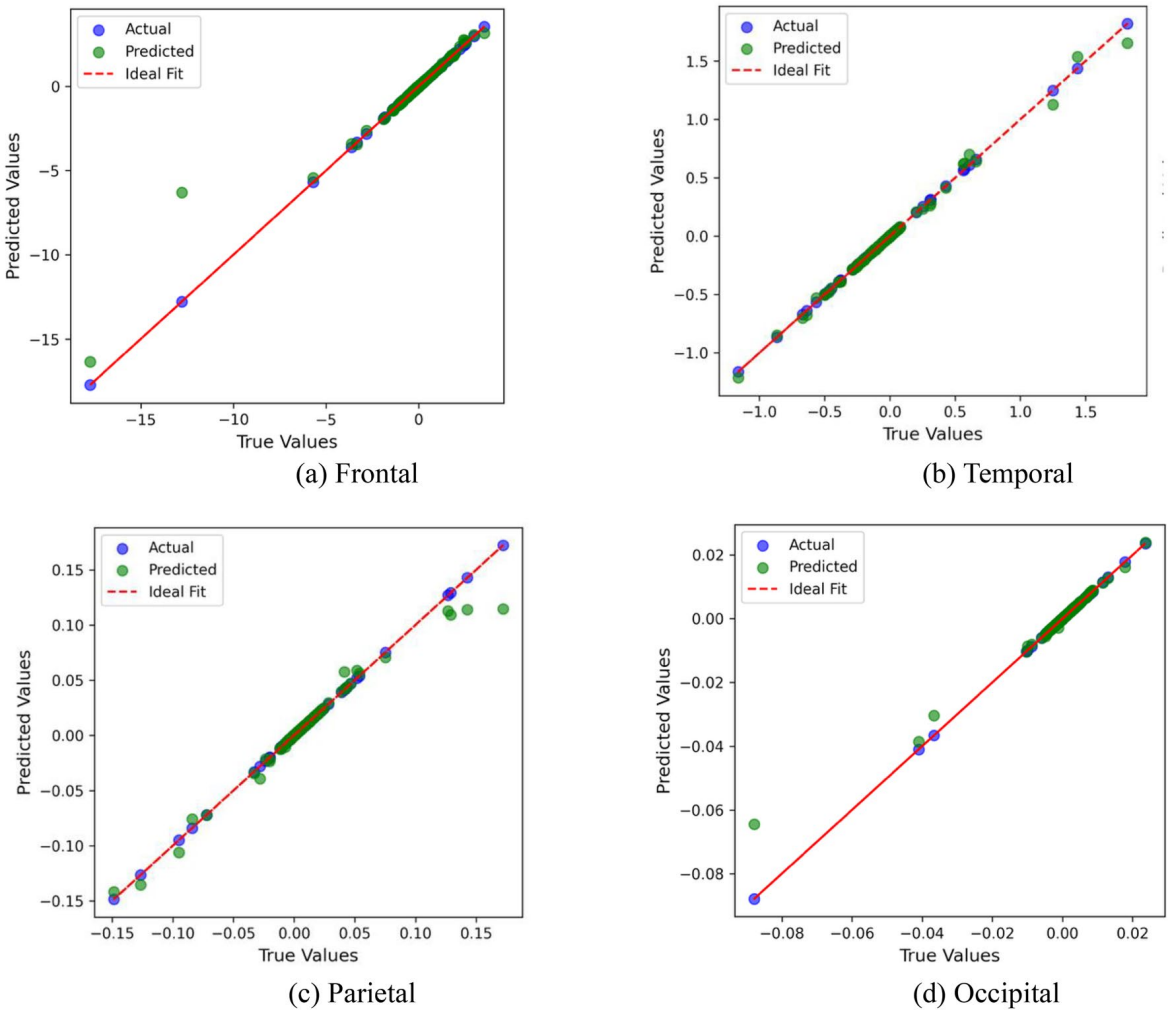
Regression performance of RFR model across different cortical areas. (**a**) Frontal cortex, (**b**) Temporal cortex, (**c**) Parietal cortex, and (**d**) Occipital cortex.

Figure 11 presents scatter plots of predicted versus actual cognitive workload values for each cortical region: Frontal (a), Temporal (b), Parietal (c), and Occipital (d). The red dashed line in each subplot denotes the ideal line of perfect prediction. Across all regions, data points are closely clustered along this line, indicating low deviation between predicted and actual values.

**Fig. 12:**
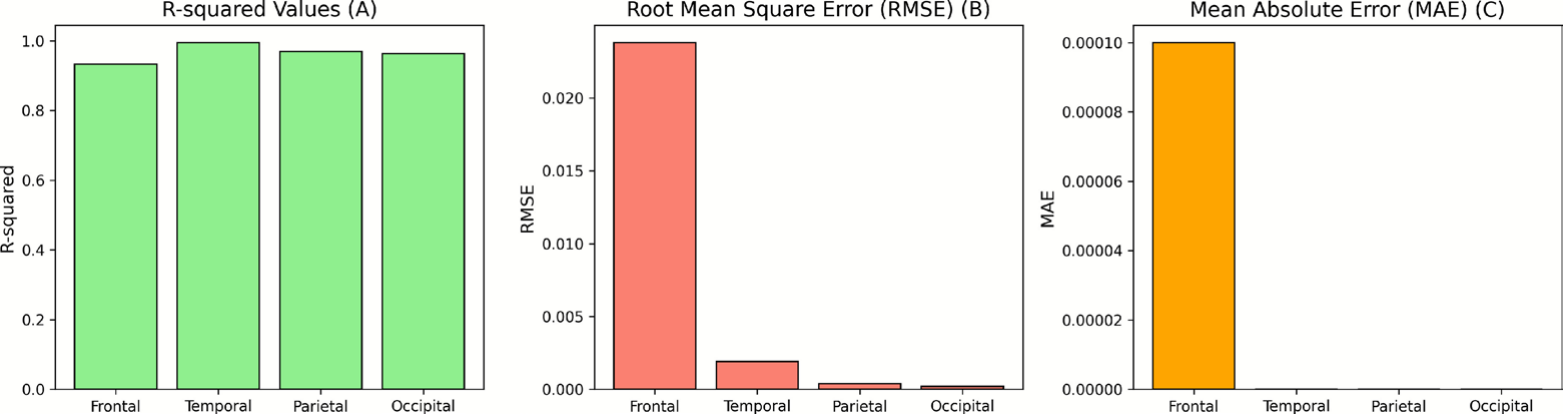
Cortical area analysis metrics: (**a**) R^2^, (**b**) RMSE, (**c**) MAE value across 4 cortical area.

Figure 12 shows comparative bar plots for R^2^ (a), RMSE (b), and MAE (c) across the four cortical regions. R^2^ values exceeded 0.93 for all regions, with the Temporal cortex achieving the highest value of 0.9951. RMSE values were lowest for the Temporal, Parietal, and Occipital cortices, while the Frontal cortex displayed a slightly higher RMSE. MAE values remained low across all regions, indicating minimal absolute error.

### Train-test split validation

A stratified 80/20 train-test split was implemented for each cortical region (Frontal, Temporal, Parietal, Occipital), with random_state = 42 to ensure reproducibility. The Random Forest Regressor (n_estimators = 100, max_depth = None, min_samples_split = 2) was trained on features extracted from 1-s EEG epochs. Model performance was evaluated using the coefficient of determination (R^2^), Mean Absolute Error (MAE), and Root Mean Square Error (RMSE) and R^2^ test values were 0.965 (Frontal), 0.995 (Temporal), 0.983 (Parietal), and 0.959 (Occipital). RMSE values were 0.0166, 0.0021, 0.0003, and 0.0002, respectively. Paired t-tests between predicted and true workload values yielded p < 0.0001 across all cortical regions. Table 4 presents the full train-test performance metrics for each region. R^2^ values ranged from approximately 0.96 to 0.999 (train) and 0.96 to 0.99 (test). MAE and RMSE values remained low across all cortical areas.

**Table 4:** Performance metrics of the random forest regressor across cortical regions using an 80/20 stratified train-test split.

Cortical Area	R^2^ (Train)	R^2^ (Test)	MAE (Train)	MAE (Test)	RMSE (Train)	RMSE (Test)	P-value Paired t-tests
Frontal	0.9654	0.9655	0.0000616	0.000112	0.0142	0.0166	< 0.0001
Temporal	0.9988	0.9948	0.0000231	0.000046	0.0010	0.0021	< 0.0001
Parietal	0.9991	0.9829	0.0000016	0.000005	0.000078	0.000270	< 0.0001
Occipital	0.9979	0.9591	0.0000006	0.000002	0.000036	0.000180	< 0.0001

### 10-Fold cross-validation performance

To rigorously evaluate model generalizability, tenfold cross-validation was applied independently to each cortical region (Frontal, Temporal, Parietal, Occipital). In this procedure, the dataset was partitioned into ten folds; in each iteration, nine folds were used for training and the remaining fold for testing, ensuring that every data point was predicted only by models that had not seen it during training. Performance was assessed using the coefficient of determination (R^2^), Mean Absolute Error (MAE), and Root Mean Square Error (RMSE). As shown in Table 5, the Random Forest Regressor achieved consistently high predictive accuracy across all cortical regions. R^2^ values exceeded 0.94 in all cases, with the Temporal cortex attaining the highest performance (R^2^ = 0.994). Error metrics were correspondingly low, with RMSE values ranging from 0.000139 (Occipital) to 0.019282 (Frontal), and MAE values remaining near zero across regions. These results confirm the robustness and stability of the proposed model when evaluated using cross-validation.

**Table 5:** 10-Fold cross-validation performance metrics of the random forest regressor across cortical regions.

Cortical Area	Number of Observations	R^2^ (tenfold CV)	MAE (tenfold CV)	RMSE (tenfold CV)
Frontal	263,221	0.9407	0.000100	0.019282
Temporal	74,941	0.9938	0.000054	0.002304
Parietal	111,675	0.9924	0.000004	0.000216
Occipital	74,450	0.9693	0.000002	0.000139

The RFR model achieved very high predictive performance across cortical regions, with R^2^ values ranging from 0.94 (Frontal) to 0.994 (Temporal), and near-zero error metrics under tenfold cross-validation Table 5. These results confirm the model’s robustness and region-specific predictive capability.

### Comparison between other regression models

A performance comparison was conducted between the RFR and three baseline regression models: SVR, Linear Regression, and Extreme Gradient Boosting (XGBoost). The results are summarized in Table 6. Across all cortical regions Frontal, Temporal, Parietal, and Occipital the RFR achieved the highest coefficient of determination (R^2^) and the lowest Root Mean Square Error (RMSE). Specifically, R^2^ values for the RFR were 0.9329 (Frontal), 0.9947 (Temporal), 0.9692 (Parietal), and 0.9635 (Occipital). In comparison, SVR and XGBoost produced negative R^2^ values in multiple regions, while Linear Regression achieved moderate performance in the Frontal cortex (R^2^ = 0.7083) but substantially lower accuracy in other areas. RMSE values for the RFR ranged from 0.0002 (Occipital) to 0.0238 (Frontal), consistently outperforming baseline models in prediction accuracy.

**Table 6:** Performance comparison of regression models across cortical regions for cognitive workload prediction.

Cortical Area	Model	R^2^	RMSE
Frontal	**Random Forest Regression**	**0.9329**	**0.0238**
	SVR	-0.0778	0.0952
	Linear Regression	0.7083	0.0495
	XGBoost	0.0193	0.0908
Temporal	**Random Forest Regression**	**0.9947**	**0.0019**
	SVR	-3.9298	0.0569
	Linear Regression	0.1097	0.0242
	XGBoost	0.3703	0.0204
Parietal	**Random Forest Regression**	**0.9692**	**0.0004**
	SVR	-451.4196	0.0494
	Linear Regression	-0.1224	0.0025
	XGBoost	0.6179	0.0014
Occipital	**Random Forest Regression**	**0.9635**	**0.0002**
	SVR	-689.7675	0.0226
	Linear Regression	-1.2158	0.0013
	XGBoost	-0.2038	0.0009

To ensure a fair and reproducible comparison among the regression models, the key hyperparameters were selected based on a combination of default settings and empirical tuning. These configurations were designed to balance predictive accuracy, resistance to overfitting, and computational feasibility. The detailed parameters are summarized in Table 7. For the RFR, the model was configured with n_estimators = 100, max_depth = None, and min_samples_split = 2, providing a balance between model complexity and efficiency. This choice is consistent with prior EEG studies showing that performance improvements plateau beyond 100 trees^82,84^. The random_state = 42 ensured reproducibility across experiments. SVR employed a radial basis function (RBF) kernel with default hyperparameters (C = 1.0, epsilon = 0.1, gamma = ’scale’), enabling non-linear mapping of EEG features. The Linear Regression model was implemented with default parameters. For XGBoost, the configuration included n_estimators = 100, max_depth = 3, learning_rate = 0.1, subsample = 0.8, and random_state = 42, aligning with commonly adopted settings that balance model performance with computational cost.

**Table 7:** Hyperparameter settings used for all regression models.

Model	Key Hyperparameters
Random Forest Regressor (RFR)	n_estimators=100, max_depth=None, min_samples_splits=2, random_state=42
Support Vector Regression (SVR)	kernel=‘rbf’, C=1.0, epsilon=0.1, gamma=‘scale’
Linear Regression	fit_intercept=True, normalize=False
XGBoost Regressor	n_estimators=100, max_depth=3, learning_rate =0.1, subsample=0.8, random_state=42

### Comparison between original articles

The predictive performance of the RFR was benchmarked against models reported by Shadpour et al.^2^, including linear regression and GLMM-LASSO approaches. RFR demonstrated superior accuracy, particularly for Matchboard Level 3 cognitive workload, achieving an R^2^ of 0.9951 in the temporal cortex with lower mean absolute error (MAE) compared to the reported range of R^2^ = 0.88–0.96 in Shadpour et al. For Matchboard Level 2, the RFR maintained an R^2^ ≈ 0.99, exceeding the GLMM-LASSO results (R^2^ = 0.94–0.97). Beyond predictive accuracy, RFR offered reduced computational complexity by relying solely on PSD-derived features, in contrast to the feature-intensive GLMM-LASSO and linear models. This efficiency enhances its feasibility for near real-time applications, underscoring RFR as a more scalable and practical approach for EEG-based cognitive workload estimation as summarized in Table 8.

**Table 8:** Comparison of predictive accuracy between this study (Random Forest Regression) and Shadpour et al. (2023).

Task/Approach	Shadpour et al. (2023)—Table 4 (Linear Random Intercept)	Shadpour et al. (2023)—Table 5 (GLMM-LASSO, Approach B)	Shadpour et al. (2023)—Table 6 (GLMM-LASSO, Approach C)	Our Study (Random Forest Regression)
Matchboard Level 3—Cognitive Workload	R^2^ = 0.88	R^2^ = 0.95	R^2^ = 0.96	**R**^**2**^** = 0.9947(Temporal Cortex)**
Matchboard Level 2—Cognitive Workload	N/A (Table 4 focuses on Level 3)	R^2^ = 0.97	R^2^ = 0.94	**R**^**2**^** ≈ 0.99(general dataset)**
Approach Overview	Linear model based on network and PSD features	High-density EEG (96 features, network + PSD)	Reduced EEG density (32 channels, 96 features)	**PSD-based features using Welch’s method**
Computational Complexity	Moderate (linear model, high-density EEG)	High (GLMM-LASSO, high-density EEG)	Moderate (GLMM-LASSO, reduced-density EEG)	**Low (focus on PSD features)**
Modeling Strength	Captures inter-regional network dynamics	Combines network flexibility and PSD features	Maintains performance with reduced EEG density	**High predictive accuracy with lower MAE**
Real-Time Applicability	Limited due to computational intensity	Limited due to high-density EEG	Moderate, reduced EEG density improves the feasibility	**High, optimized for near real-time applications**

Providing EEG data for cognitive workload assessment and performance prediction studies, a comparator analysis is presented in Table 9. Early works, such as Aricò et al.^41^, focused on signal analysis and passive BCI applications aimed at improving modeling accuracy through better signal characterization. Subsequent studies, including Borghini et al.^49^ and Salaken et al.^43^, applied machine learning techniques that enhanced classification performance, though many relied on high-density EEG datasets. More recent regression-based approaches, such as those by Shadpour et al.^2^ and Murat Teksin et al.^88^, achieved improved prediction accuracy but faced substantial computational complexity. In contrast, the present study employs Random Forest Regression on PSD-derived features, achieving a predictive accuracy of R^2^ = 0.9947 while maintaining computational efficiency suitable for real-time cognitive workload assessment.

## Discussion

This study demonstrates that RFR, when applied to PSD and time-domain EEG features, can achieve high predictive accuracy in CW estimation across multiple cortical regions. The model yielded R^2^ values exceeding 0.93 in all regions, with the temporal cortex attaining the highest accuracy (R^2^ = 0.9947) and the lowest error metrics. This result reinforces the temporal cortex’s established role in workload processing, consistent with previous findings identifying it as a hub for integrating auditory, perceptual, and mnemonic functions under cognitively demanding conditions^46^. Compared to baseline models, including SVR, Linear Regression, and Extreme Gradient Boosting (XGBoost), the RFR exhibited superior generalization, as evidenced by lower RMSE and MAE values. The ensemble-based architecture effectively captured nonlinear EEG-CW relationships while reducing overfitting through aggregation of multiple decision trees, aligning with prior studies advocating tree-based ensemble methods for high-dimensional neurophysiological datasets^75,82,84^.

While the model achieved unusually high R^2^ values (up to 0.994), which exceed many prior EEG workload studies, these findings should be interpreted with caution. The modest sample size and reliance on a task-specific dataset may partially account for the strong performance. Future validation on larger and more heterogeneous datasets will be essential to establish the robustness and generalizability of the proposed approach. The model’s superior performance can be attributed to three primary factors: (1) rigorous preprocessing, including bandpass filtering, independent component analysis (ICA) for artifact removal, and Welch’s method for PSD estimation; (2) targeted feature selection emphasizing RMS, kurtosis, and PSD features within theta and beta bands; and (3) the ensemble learning design of RFR, which improves robustness and accuracy by mitigating overfitting and effectively modeling non-linear feature interactions. From a neurophysiological perspective, the observed dominance of RMS, kurtosis, and skewness as predictive features highlights their sensitivity to amplitude fluctuations, burst-like neural events, and waveform asymmetries factors closely associated with cognitive effort, attentional load, and stress^31,89^ . Region-specific trends, such as elevated kurtosis in the temporal cortex and strong RMS contributions in parietal and occipital cortices, likely reflect task-specific sensory processing and visuospatial integration demands.

Comparative literature analysis Table 9 indicates that earlier studies, such as those by Aricò et al., 2016 and Borghini et al., 2017, predominantly employed classification-based approaches for mental state prediction using passive brain-computer interfaces (BCIs) or support vector machines. More recent regression-based works, including Shadpour et al., 2023 and Teksin et al.,2024, have demonstrated the feasibility of EEG-based surgical performance prediction but faced computational challenges and limited real-time capability, particularly with high-density EEG. In contrast, the present framework leverages PSD-based features with reduced computational complexity, enabling rapid inference without compromising accuracy. As shown in Tables 7 and 8, the RFR consistently outperformed SVR, Linear Regression, and XGBoost in both accuracy and computational efficiency. Unlike GLMM-LASSO models, which require high-density EEG and complex connectivity metrics, the proposed approach achieves comparable or superior accuracy with substantially lower computational cost, supporting its feasibility for near real-time applications. Furthermore, region-specific modeling shows promise in reducing the number of EEG channels required, thereby enhancing system portability for neuroergonomics applications in domains such as robot-assisted surgery, aviation, immersive training, and military decision-making.

**Table 9:** Literature review of EEG-based cognitive workload evaluation and performance prediction.

Author(s)	Focus Area	Methodology	Key Findings	Limitations	Ref
Aricò et al., 2016	Passive brain-computer interfaces (BCIs) for mental state classification	Explored passive BCIs using EEG features for classification	Achieved ~ 80% accuracy in mental state classification	Task-specific BCIs; are not robust for generalized cognitive workload evaluation	^41^
Borghini et al., 2017	Classification of EEG features for cognitive workload and mental state analysis	Applied SVM and KNN for feature classification	Achieved ~ 85% accuracy in classification tasks	Limited dataset diversity; did not address dynamic network flexibility	^49^
Salaken et al., 2020	Cognitive workload prediction using machine learning	Applied SVM and RF models for workload classification	Achieved ~ 88% accuracy in workload classification	Limited exploration of feature engineering and dataset size	^43^
Zhang et al., 2020	EEG classification using random forest	Focused on feature extraction and classification using RF models	Achieved ~ 92% classification accuracy	Did not integrate time–frequency domain features like PSD	^15^
Lin et al., 2021	Temporal EEG feature analysis for workload prediction	Implemented LSTM and deep learning models for time-series EEG data	Achieved ~ 88% accuracy in workload classification	Requires large datasets and high computational power	^46^
Shadpour et al., 2023	Surgical performance evaluation using EEG data and machine learning	Used GLMM-LASSO and linear models for regression analysis	Achieved R^2^ = 0.97; highlighted the importance of EEG data for performance evaluation	High computational demands; limited real-time applicability; reliance on high-density EEG data	^2^
Murat Teksin et al., 2024	Performance prediction in robot-assisted surgery using eye-tracking and ML	Used XGBoost regression for performance prediction based on eye-tracking features	Achieved R^2^ = 0.456 for regression; demonstrated potential of eye-tracking in performance analysis	Relied solely on eye-tracking data; did not incorporate EEG or pupillometry in depth	^88^
This Study	Cognitive workload evaluation using EEG and ML	Applied Random Forest Regression with PSD and time–frequency features for workload evaluation	Achieved R^2^ = 0.9947 for cognitive workload prediction; optimized for near real-time monitoring	Limited exploration of functional brain network features	

## Conclusions

This study demonstrates the effectiveness of machine learning models, particularly RFR, in estimating cognitive workload from EEG signals during RAS. Leveraging epoched time and frequency domain features, the proposed approach achieved R^2^ values exceeding 0.93 across all cortical regions, peaking at 0.9947 in the temporal cortex. Compared with more computationally intensive frameworks, such as those employing graph-theoretic or connectivity-based features (e.g., GLMM-LASSO), the RFR model offers an interpretable and resource-efficient solution. Robustness was confirmed via tenfold cross-validation, with all results achieving strong statistical significance (*p* < 0.0001). Benchmarking against baseline models, including SVR, Linear Regression, and XGBoost, demonstrated RFR’s superior performance through consistently lower RMSE and higher R^2^ values across all brain regions. These results underscore the model’s suitability for EEG-based CW estimation in high-stakes operational environments.

Despite its strengths, the current framework does not incorporate functional connectivity metrics or network-based representations, which could yield deeper neurophysiological insight. Future work should explore integrating such features to enhance both interpretability and predictive accuracy. Expanding the dataset to include larger and more diverse participant cohorts, and validating real-time performance on embedded high-density EEG systems, will be critical for clinical and operational adoption. Overall, the proposed RFR framework establishes a new performance benchmark for EEG-based CW modeling in surgical contexts, offering a scalable, interpretable, and computationally efficient foundation for advancing neuroergonomics, cognitive state monitoring, and performance optimization in complex real-world tasks.

## Limitations and future work

While the proposed RFR model demonstrated high predictive accuracy for CW estimation using EEG-derived features, several limitations should be acknowledged to contextualize the findings and guide future improvements. First, the dataset used in this study was obtained from a publicly available, task-specific EEG dataset with a relatively modest sample size (n = 25). Although cortical stratification and task variation were incorporated, the limited demographic and experiential diversity (e.g., age, surgical experience) may constrain the generalizability of the model’s predictions across broader surgical or operational domains. Future studies should include larger, multi-center cohorts with greater inter-individual variability to enhance the external validity and robustness of the model in clinical and real-world settings. Second, the preprocessing pipeline employed downsampling from 500 to 128 Hz to reduce computational complexity. Although topographical consistency and frequency-domain characteristics were retained as supported by visual analyses of spectral maps and time–frequency spectrograms fine-grained high-frequency oscillations (e.g., upper gamma band > 50 Hz) may have been attenuated or lost. Future work should systematically evaluate the trade-offs between computational efficiency and spectral fidelity in downsampling strategies.

Third, this study utilized a Common Average Reference (CAR) montage to improve signal-to-noise ratio and maintain standard EEG preprocessing protocols. However, CAR may introduce spatial smearing in high-density EEG recordings. More advanced referencing techniques, such as the surface Laplacian or the Reference Electrode Standardization Technique (REST), can provide better spatial resolution and should be explored in future research for improved localization and interpretability. Fourth, although robust time and frequency domain features were extracted, the model did not incorporate functional brain network features such as coherence, phase-locking value, or graph-theoretic measures. These network-level metrics have been shown to capture the dynamic reconfiguration of brain connectivity during cognitive tasks and may offer complementary information to power-based features. Integrating such measures could improve both model performance and neuroscientific insight.

Fifth, while the Random Forest Regression model achieved very high predictive accuracy, with R^2^ values exceeding 0.99 in some regions, such performance is uncommon in EEG workload studies. Although tenfold cross-validation was employed to mitigate overfitting, these results should be interpreted cautiously, and validation on larger, independent datasets will be essential to confirm generalizability. Finally, although the study evaluated statistical performance metrics such as R^2^, RMSE, and MAE with tenfold cross-validation, it did not assess real-time processing constraints such as latency, throughput, or memory usage. For practical deployment in surgical or neuroergonomic environments, these system-level metrics should be benchmarked to support low-latency, real-time cognitive workload monitoring systems.

## Data Availability

The EEG and eye-gaze data analyzed in this study are publicly available from the PhysioNet repository under the title “Electroencephalogram and Eye-Gaze Datasets for Robot-Assisted Surgery Performance Evaluation” (version 1.0.0) (https://www.physionet.org/content/eeg-eye-gaze-data/1.0.0/). The dataset was originally collected and published by Shafiei et al. (2023) and is accessible via PhysioNet with 10.13026/qj5m-n649 (RRID: SCR_007345). The data are distributed under the PhysioNet Credentialed Health Data License. No new human subject data were collected for this study.

## Code availability

The custom Python code underlying EEG preprocessing, epoch-based feature extraction, and Random Forest Regression modeling used in this study has been made publicly available. The code is archived with a permanent DOI via Zenodo: 10.5281/zenodo.18096105 and maintained in the GitHub repository https://github.com/mohatheef/eeg-cognitive-workload-rfr researchers may access, review, and reuse the code in accordance with the repository license.
